# 125. Induction of Resistance Against Antipseudomonal Agents: Comparison of Novel b-Lactam/b-Lactamase Inhibitor (BL/BLI) Combinations and Other b-lactam Agents

**DOI:** 10.1093/ofid/ofac492.203

**Published:** 2022-12-15

**Authors:** Mariana Castanheira, Jill Lindley, Timothy Doyle, John H Kimbrough, Jessica Ewald, Helio S Sader

**Affiliations:** JMI Laboratories, North Liberty, Iowa; JMI Laboratories, North Liberty, Iowa; JMI Laboratories, North Liberty, Iowa; JMI Laboratories, North Liberty, Iowa; JMI Laboratories, North Liberty, Iowa; JMI Laboratories, North Liberty, Iowa

## Abstract

**Background:**

The acquisition of mutations is the main driver of β-lactam resistance in *Pseudomonas aeruginosa* isolates. New BL/BLIs, such as ceftazidime-avibactam (CAZ-AVI), ceftolozane-tazobactam (C/T), and imipenem-relebactam (IMI-REL), are active against most *P. aeruginosa* isolates from US hospitals, but the ability of these agents to induce resistance have not been explored. We subjected 8 *P. aeruginosa* isolates, including ATCC 27853 and 7 clinical isolates, to a 10-day serial passage with 6 antipseudomonal agents to evaluate resistance levels and mechanisms in terminal mutant strains.

Fold change in MIC results from parent isolate to terminal mutant

**Figure:**
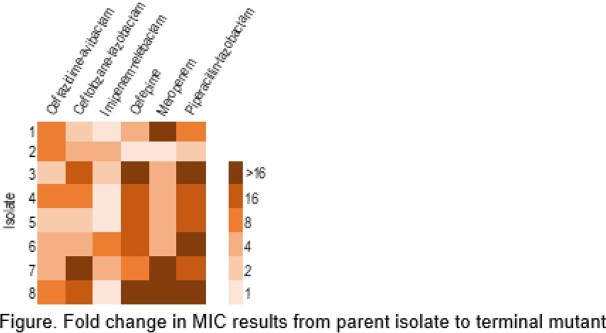

**Methods:**

Serial passaging was performed in broth microdilution (BMD) for CAZ-AVI, IMI-REL, C/T, meropenem (MEM), cefepime (FEP), and piperacillin-tazobactam (P/T). The MIC of the terminal mutants was determined after 2X passaging on drug-free agar. Parent strains and terminal mutants were subjected to short-read whole genome sequencing (WGS) at 100X coverage. Parent isolates were sequenced using long-read WGS and the data was combined with short-reading sequencing for single nucleotide polymorphism (SNP) analysis.

**Results:**

Overall, IMI-REL (1- to 4-fold) and CAZ-AVI (2- to 8-fold) displayed lower fold increases in MIC values when compared to other agents tested (Figure). Of the CAZ-AVI terminal mutants, 3 displayed a *nalD* regulator alteration, and 1 of these had a *clpA* chaperone missense substitution. FEP terminal mutants exhibited alterations in *ampD*, *mexB*, and the TetR family transcriptional regulator AmrR. C/T mutants had *ampG* and *ftsI* missense alterations. MEM mutants had *nalC, ftsI*, and *phoP* missense alterations. Mutations in *merR, nalC,* and *ampD* were observed in the P/T terminal mutants. Among 2 IMI-REL terminal mutants displaying a SNP alteration, 1 displayed a nonsense mutation in *pilF*, a pilus forming protein. Many terminal mutants displayed alterations in genes not commonly associated to β-lactam resistance.

**Conclusion:**

MEM, FEP, and P/T terminal mutants displayed high MIC values compared to those obtained after exposure to C/T, CAZ-AVI, and IMI-REL. This data might indicate a benefit of using these newer agents to prevent the emergence of high-level resistance.

**Disclosures:**

**Mariana Castanheira, PhD**, AbbVie: Grant/Research Support|Cidara: Grant/Research Support|GSK: Grant/Research Support|Melinta: Grant/Research Support|Pfizer: Grant/Research Support|Shionogi: Grant/Research Support **Jill Lindley, BS**, AbbVie: Grant/Research Support **Timothy Doyle, MS**, AbbVie: Grant/Research Support **John H. Kimbrough, PhD**, AbbVie: Grant/Research Support|GSK: Grant/Research Support **Jessica Ewald, PhD**, AbbVie: Grant/Research Support **Helio S. Sader, MD, PhD**, AbbVie: Grant/Research Support|Cidara: Grant/Research Support|Melinta: Grant/Research Support|Nabriva Therapeutics: Grant/Research Support|Pfizer: Grant/Research Support.

